# Theory of Mind and Preference Learning at the Interface of Cognitive Science, Neuroscience, and AI: A Review

**DOI:** 10.3389/frai.2022.778852

**Published:** 2022-04-05

**Authors:** Christelle Langley, Bogdan Ionut Cirstea, Fabio Cuzzolin, Barbara J. Sahakian

**Affiliations:** ^1^Department of Psychiatry, University of Cambridge, Cambridge, United Kingdom; ^2^School of Engineering, Computing and Mathematics, Oxford Brookes University, Oxford, United Kingdom

**Keywords:** human theory of mind, machine theory of mind, artificial intelligence, cognitive and neuroscience, inverse reinforcement learning

## Abstract

Theory of Mind (ToM)—the ability of the human mind to attribute mental states to others—is a key component of human cognition. In order to understand other people's mental states or viewpoint and to have successful interactions with others within social and occupational environments, this form of social cognition is essential. The same capability of inferring human mental states is a prerequisite for artificial intelligence (AI) to be integrated into society, for example in healthcare and the motoring industry. Autonomous cars will need to be able to infer the mental states of human drivers and pedestrians to predict their behavior. In the literature, there has been an increasing understanding of ToM, specifically with increasing cognitive science studies in children and in individuals with Autism Spectrum Disorder. Similarly, with neuroimaging studies there is now a better understanding of the neural mechanisms that underlie ToM. In addition, new AI algorithms for inferring human mental states have been proposed with more complex applications and better generalisability. In this review, we synthesize the existing understanding of ToM in cognitive and neurosciences and the AI computational models that have been proposed. We focus on preference learning as an area of particular interest and the most recent neurocognitive and computational ToM models. We also discuss the limitations of existing models and hint at potential approaches to allow ToM models to fully express the complexity of the human mind in all its aspects, including values and preferences.

## Introduction

### Theory of Mind as a Cognitive Process

There is a greater and greater reliance on artificial intelligence (AI) in many different aspects of life. Two very prominent areas include autonomous cars and physical and mental health care. For these applications of AI it is essential that they include a component of Theory of mind (ToM; Cuzzolin et al., 2020). ToM is the ability of the human mind to attribute mental states to others and is a key component of human cognition. In order to understand other people's mental states or viewpoint and to have successful interactions with others within social and occupational environments, this form of social cognition is arguably essential. It is also beneficial for individual and societal wellbeing to feel that your thoughts and emotions have been understood by others, thereby promoting positive social engagement.

### “Hot” and “Cold” Cognition

This task, however, is extremely challenging, as ToM is a holistic process that can be decomposed into a number of separate *hot* and *cold* cognitive processes. Cold cognitive processes are non-emotional processes, whereas hot cognition relates to social and emotional cognition (Roiser and Sahakian, 2013). Social cognition is the way in which people process, remember, and use information in social contexts to explain and predict their own behavior and that of others. Hot and cold cognition plays a role in risky decision-making, which may become important for many human-AI interactions including in autonomous cars or surgical robots (Lawrence et al., 2008).

### Psychological Approaches to Understanding Theory of Mind

To understand the mental states of others it is likely that there are individual differences in the cognitive strategies that we use. These strategies are likely to have developed during childhood and adolescence, particularly during ages 1–5 (Frith and Frith, 2005; Academy of Medical Sciences, 2019). To some extent, the hot and cold processes of the ToM strategies that will be most successful are context dependent. Examples of different strategies for ToM are the theory-theory approach (Gopnik and Wellman, 1992) and the simulation-theory approach (Gordon, 1986). The former can be based on a set of innate rules or on causal and probabilistic reasoning models, whereas the latter is more of a perspective based approach. Interestingly, the theory-theory approach may be analogous to cold cognition where intellectual processes are used to infer mental states, whereas the simulation-theory approach is more of a hot technique, which relies on one's own motivations and reasoning ability (Gordon, 1996). This is not to suggest that either strategy solely relies on hot or cold cognition, as there is an important interaction between the two. Additionally, in understanding ToM it is important to evaluate the genetic, hormonal and environmental contributions and their interactions. Furthermore, it may be that these influences vary in healthy individuals and those with psychiatric disorders, such as Autism Spectrum Disorder (ASD).

As AI becomes more readily used in society, it is important to ensure that AI and human interactions, be it in the motoring industry, such as autonomous cars, or healthcare including surgical robots or mental health treatment and management, including in depression and dementia, are optimally beneficial. This would require AI models to have ToM abilities. Therefore, this review aims to synthesize what we currently know about human ToM and machine ToM, with the intention to stimulate discussion to better integrate the two fields. It appears thatAI has not yet provided a truly holistic approach to ToM, and has rather focused on separate components, perhaps because these are easier to model. In keeping with the aim of this review, we briefly present selected cognitive and neuroimaging research that has contributed to our understanding of ToM. Furthermore, we attempt to synthesize the existing neurocognitive and AI models that have been proposed, and critically discuss if and where potentially useful findings from cognitive and neurosciences have found their way into computational models. Finally, we provide suggestions for future research to create computational and AI models that are heavily guided by knowledge and data from cognitive and neuroscience research. The aim is to facilitate discussion as to how ToM can be holistically integrated into AI models in the context of AI applications in healthcare, automated driving and the service industry where AI will interact closely with humans.

## Cognitive and Neural Mechanisms of Theory of Mind

ToM is a key component of human cognition. The term was first used by Premack and Woodruff (1978), who were attempting to determine whether a chimpanzee had ToM. Subsequent research suggested a *social brain* hypothesis, where authors argued that, from an evolutionary standpoint, having good ToM abilities would be beneficial for increasingly complex social environments (Brothers, 1990). Cognitive and neuroimaging studies have examined ToM abilities in not only normal developing children and healthy adults, but also in a number of neurodevelopmental and neuropsychiatric disorders. Research on individuals with ASD, where a ToM deficit is present, has particularly contributed to our understanding of human ToM. Given the knowledge we have acquired about human ToM through cognitive science and neuroscience it could prove advantageous for machine ToM to utilize this knowledge. It should be stated that not all human ToM aspects are currently transferable to machine ToM with the technological limitations.

### Development of Theory of Mind

We review the literature related to the development of theory of mind which can help AI researchers to understand the learning phases of ToM skills and strategies. While it is typically considered that by the age of 5 children have developed many aspects of ToM, studies have shown that some ToM abilities are established very early in life, whereas others develop slightly later. Baron-Cohen (1995) described a developmental model of ToM. From around 6 months of age human infants begin to distinguish between the motion of inanimate and animate objects. At 12 months of age joint attention is developed, where the infant has the cognitive capacity to represent its own perception, that of an agent (e.g., mother) and that of an object. By 14–18 months, through gaze direction, the infant begins to understand the mental states of desire, intention and the causal relation between emotions and goals (Saxe et al., 2004). Liszkowski et al. (2006) showed that children as young as 12–18 months were able to infer an adult's behavior and aid them. In this particular experiment, infants watched an adult write with a marker on a piece of paper. The marker would drop off the table, not seen by the adult. When the adult began randomly searching for the marker, the infant would either point to or retrieve the marker, ignoring any other distractors. Between 18 and 24 months toddlers begin to distinguish between real and pretend events and often start to engage in pretend play around this age. Around the age of 3–4 children begin to understand the differences between their own and others' beliefs and knowledge, thereby beginning to comprehend false beliefs, but this ability does not become fully stable until age 5–6. Understanding metaphors, irony and sarcasm only establishes around age 6–7.

There is considerable individual variability in the development of ToM. This may be due to genetic, hormonal and environmental influences as well as their interaction. Interestingly, a genetic study comparing 1,116 monozygotic and dizygotic 5 years old twins found that ToM ability was more attributable to environment than genes (Hughes et al., 2005). However, a previous smaller scale study of 3-year-old twins did show some genetic influence on ToM abilities (Hughes and Cutting, 1999). Environmental factors such as regular exposure to language and conversations about mental states are associated with better development of ToM abilities (Dunn and Brophy, 2005). McAlister and Peterson (2006) found that preschool aged children with siblings at home had better ToM abilities compared to only children with no siblings. Similarly, children whose parents frequently refer to mental states when talking to them develop ToM abilities at an earlier age (Carpendale and Lewis, 2004). Moreover, the development of ToM is strongly linked to the development of language (Frith and Frith, 2003; de Villiers, 2007; de Villiers et al., 2014; Kim, 2020) as well as executive function to some extent (Arslan et al., 2017a; Kim, 2020). Nevertheless, a strong genetic link exists for ASD (Huguet et al., 2016) and there is evidence of an association between fetal hormonal testosterone and autistic traits (Auyeung et al., 2009). This potentially suggests that there are different influences on ToM abilities between typically developing and non-typically developing children. Cognitive studies of ToM abilities have highlighted that there is a strong learning component, through environment, for developing ToM abilities; this is largely overlooked in current AI models. The development of ToM in humans highlights the fact that all ToM abilities are not developed at the same time: for as such the importance of continual learning is a crucial aspect for developing a machine ToM. Similarly, ToM abilities develop in social contexts in humans and through the development of language; as such, the impact of artificial and isolated learning settings in AI should be considered.

Higher-order ToM abilities also exist, and are established later in the developmental trajectory. Second-order ToM involves predicting what one person thinks or feels about what another person is thinking or feeling (Westby and Robinson, 2014). The literature shows that even humans struggle with higher-order ToM abilities (Kinderman et al., 1998; Hedden and Zhang, 2002; Flobbe et al., 2008; Meijering et al., 2010, 2014). This has potential implications for machine ToM as AI becomes increasingly used in society. However, in this review we focus on first-order ToM abilities.

### Dysfunction of Theory of Mind

There are several instances where ToM is disrupted, for example in ASD. An understanding of the cases in which humans display poor ToM abilities may be useful to guide AI in developing models with good ToM abilities. Individuals with ASD actively avoid eye contact, engage in stereotyped or repetitive behaviors and struggle to establish emotional relationships. There is a large amount of research on impaired ToM abilities in ASD, which is not within the scope of this review, but it is important to note that individuals with ASD have difficulty with tasks of ToM. In high functioning ASD this is often independent of intelligence (IQ) and other cognitive capacities that remain intact (Baron-Cohen, 1995). Senju et al. (2010) showed that 6–8 year old children with ASD were unable to anticipate an actor's behavior based on his false belief. They used a false belief task where an actor placed a toy in a box, when the actor was not looking the toy was moved. Children were asked to anticipate where the actor would look and were unable to correctly do so, whereas typically developing children as young as 25 months are able to correctly anticipate the actor's behavior in the same task (Southgate et al., 2007). Children with attention deficit hyperactivity disorder (ADHD) often have impaired attention and executive functions, but ToM abilities remain intact (Kain and Perner, 2003) suggesting that ToM is independent of other cognitive functions. Executive functions are higher-level cognitive processes, such as planning and problem-solving, and are normally subserved by frontal lobe networks. Research on individuals with ASD has demonstrated that they may struggle to understand even their own intentions and the relationship between this and understanding others intentions needs to be further elucidated. This further demonstrates the importance of understanding the self in computational ToM. Indeed, “perspective taking” (“what would I do / think / feel if I were in his/her situation?”) in an internal simulation process plays a significant role in ToM in humans (Barnes-Holmes et al., 2004).

Although ASD is the classic example of disrupted ToM abilities, there is evidence of impaired ToM in disorders such as schizophrenia (Brüne, 2005), bipolar disorder (Kerr et al., 2003), frontal lesions following stroke (Happé et al., 1999), and even in aging (Phillips et al., 2002).

### Assessing Theory of Mind

ToM is a complex psychological process with multiple components; as such, multiple tasks for assessing various aspects of ToM have been developed. Baron-Cohen (2000) provides a review of the early tasks assessing various aspects related to ToM. Here we provide only a brief summary. ToM has been well-studied in humans in cognitive and neuroscience, given this wealth of information, it would make sense to look to how to integrate some human approaches to ToM to machine ToM. Understanding how ToM is assessed in humans, may provide not only a better understanding of how to train ToM in AI models, but also ways to test computational models of AI. Tasks assessing the *mental-physical* distinction involved one character that was having a mental experience (thinking about a dog) and another character having a physical experience (holding a dog). The subject is then asked to judge whether the character is able to perform a specific task (e.g., which character can stroke the dog?). Typically developing children aged 3–4 were able to accurately distinguish mental and physical actions (Wellman and Estes, 1986), whereas children with ASD were significantly impaired (Baron-Cohen, 1989). Flavell et al. (1986) demonstrated that children from the age of 4 are able to distinguish *appearance from reality*. When presented with a candle in the shape of an apple, they accurately state that the object is a candle. On the other hand children with ASD mistakenly judge the object as an apple (Baron-Cohen, 1989). *First-order false belief* tasks involve inferring a single person's mental state and understanding that people can have different thoughts about the same situation. The classic “Sally-and-Anne Test” (Wimmer and Perner, 1983) is a test of first-order false belief. In this task an object is hidden by one character (Anne) in the absence of a second character (Sally). The key question is where Sally would look for the object when she returned: either the location it was before she left the scene, or the place where Anne had moved it. Wimmer and Perner (1983) showed that typically developing 4 year olds were able to understand others' perspectives. Again, children with ASD are unable to shift their perspective to what another person may think, and simply report what they believe (Baron-Cohen et al., 1985, 1986). The Sally-and-Anne Test is a seminal ToM test in human ToM, as such it could be useful to use this task to test the ToM abilities of machine ToM. However, it should also be noted that this is an early stage of ToM development, and the models may not be as sophisticated as adult ToM. Nevertheless, this seems like a potential first step.

Another key component of ToM is being able to understand how knowledge is acquired to appreciate what an individual does or does not know. One example is that children are given a story where one character only touches a box, whereas another character looks inside the box. Typically developing 3 year olds are able to identify which character knows what is in the box (Pratt and Bryant, 1990), whereas children with ASD cannot (Leslie and Frith, 1988; Baron-Cohen and Goodhart, 1994). Children with ASD also struggle to understand their own intentions. Phillips et al. (1998) developed a novel test where children were asked to shoot a toy gun at one of six targets and state their intended target. The experimenter would manipulate the outcome; this was not known by the child. Typically developing 4 year olds were able to correctly state their intended outcome, even if the actual outcome differed, whereas children with ASD answered with the actual outcome regardless of their intended target. This work further demonstrates the importance of self for ToM. Additionally, tasks like these could help to test whether the machine models of ToM, indeed have ToM abilities, or have simply learned an intended target or outcome.

Castelli et al. (2002) developed a novel task where the animations were not human, but rather participants were presented with animations of two moving triangles. Each animation was manipulated to show random, goal-directed or mentalising movements. Individuals with ASD made fewer and less accurate interpretations of the animations that were related to mentalising (e.g., two triangles bounced up and down happily; Baron-Cohen, 2000), thereby suggesting that a ToM is not only related to human interactions. This is an important finding for AI, as it demonstrates that ToM is not only related to human interactions, but may be attributed to objects and machines, e.g., in autonomous cars and robots.

ToM is more broadly incorporated as a key component of social cognition. As such tests of emotion recognition, social cooperation and morality have been established to assess broader areas of ToM and social cognition. There is a strong correlation between ToM and emotion recognition (Mier et al., 2010). There are a number of tests that assess emotion recognition, many of which require the identification of basic emotions, such as happiness and sadness. Some use only the eyes for stimuli, whereas others include the full face. Indeed a meta-analysis found that emotion recognition was impaired in ASD (Uljarevic and Hamilton, 2013). There has been significant work in AI models for emotion recognition, but as discussed previously and highlighted by the number of different tasks, ToM goes beyond simple emotion recognition, and more work is required for machines to truly develop ToM abilities.

A well-known test of social cooperation is the Prisoner's Dilemma (see Algarni, 2017). Here we do not go into detail on the literature regarding this task; although there seem to be strong links between cooperation and deception behavior and the cognitive capacity to infer the mental states of putative allies or competitors. Sally and Hill (2006) showed that younger children performed worse compared to older children on tasks of social cooperation; children with ASD also performed worse. Li et al. (2014) found an interesting relationship between judgements of morality and cooperative play. Both typically developing children and children with ASD made correct moral judgements. However, typically developing children changed their cooperative behavior depending on whether they were interacting with the morally nice or morally naughty child, whereas ASD children did not adjust their cooperative behavior. Again, this task may be useful to test ToM abilities in machines. If they perform more like an ASD child, we know that further work is required for the model. These tasks cover more complex forms of ToM than emotion recognition, but are important for developing “holistic” models. However, it is likely that these more complex forms of ToM are not yet within the technological abilities of machine ToM.

In terms of moral judgement, tasks have been developed to assess an individual's moral reasoning. An example is the moral judgement task developed by Bland et al. (2016). This task consists of cartoons of various scenarios: e.g., liquid is dropped onto a computer. The subject is asked to judge the amount of shame, guilt and annoyance and how bad they would feel if in the situation portrayed by the cartoon. The same cartoon is displayed where the subject is the agent or victim and the action is deliberate or accidental. A systematic review of morality in ASD interestingly showed that individuals with ASD made morally correct judgements, but that their moral reasoning was compromised, thereby suggesting that they intuitively knew what was moral, but not why (Dempsey et al., 2020). This is an important finding for machine ToM, as to fully have ToM abilities we must also be able to understand why. The current benchmarks for machine ToM largely overlook these elements and this is where knowledge of human ToM could be key for machine ToM.

In summary, there are a number of validated cognitive tasks available to assess various aspects of ToM. Nevertheless, the interaction between these aspects has not been fully explored. The variety of tasks available to assess ToM in humans has an important implication for AI research which has often focused on specific subcomponents and has not considered a more holistic approach.

### Neural Mechanisms of Theory of Mind

Here we briefly review some of the neuroimaging studies of ToM, an understanding of the neural basis of ToM may be of particular importance for developing new AI models incorporating ToM. Neuroimaging research has shown that ToM involves a number of brain regions in the frontal, temporal and parietal lobes. More specifically, studies have found that the medial prefrontal cortex (mPFC), anterior cingulate cortex (ACC), posterior cingulate cortex (PCC), precuneus, temporo-parietal junction (TPJ), middle temporal gyrus, and superior temporal sulcus are all involved in tasks that require ToM (Saxe et al., 2004; Amodio and Frith, 2006). Studies have suggested that the mPFC and ACC are involved in distinguishing self from other and reality (deception), as well as being related to error monitoring and saliency (Schlaffke et al., 2015). The precuneus is associated with the experience of agency, and the TPJ is involved in the representation of others' thoughts (Schlaffke et al., 2015). Due to the complex nature of ToM and the number of different aspects involved, there is some heterogeneity in the neural mechanisms underlying ToM.

Schurz et al. (2014) conducted an important meta-analysis of neural mechanisms in ToM, but rather than pooling across studies, they specifically examined different task groups to identify regions that were commonly activated, or more specific to certain task groups. Indeed, they found a core network involved in all ToM tasks that included the mPFC and TPJ, suggesting that these regions are key to ToM functioning (Schurz et al., 2014). While this core network was consistently activated, each task group showed more specific regions that were involved. In the false belief task group regions including the middle temporal gyrus, inferior parietal lobe, precuneus, ACC, PCC, and insula were activated in addition to the mPFC and TPJ. For the trait judgement task group (tasks that involve information about traits of a person) there was activation of the PCC, precuneus, temporal-parietal cortices, and the anterior temporal lobes. In the strategic games task group the largest activation was in the mPFC, extending to the ACC and the posterior frontal cortex. There was additional activation in the thalamus, middle temporal area and left fusiform gyrus. For social animations, the largest area of convergence was in the temporo-parietal cortices, but also included the thalamus, inferior frontal gyrus, anterior temporal lobe, and cerebellum. For tasks associated with social over general intelligence (“mind in the eyes”), regions of convergent activation included the inferior frontal gyrus, precentral and middle frontal gyri and the insula. There was also some activation in the fusiform gyrus. For tasks involving rational actions regions such as the precuneus, PCC and anterior temporal lobe were activated in addition to the TPJ and mPFC. These findings suggest that while there are distinct brain regions involved in specific tasks, there seems to be a core network involved across all ToM tasks. Thereby, suggesting that humans do employ a more holistic approach to ToM. This is an important finding for machine ToM, where the holistic element is largely overlooked.

The findings from neuroimaging research have highlighted an interesting dissociation between *cognitive* and *affective* ToM. Cognitive ToM is related to the ability to represent thoughts, intentions and beliefs, whereas affective ToM is more associated with the representation of emotional states and feelings. The Schurz et al. (2014) meta-analysis did not identify the amygdala as an area of interest, but it is a key region in emotion recognition. Indeed, when examining cognitive ToM tasks the amygdala does not play a critical role, whereas it is strongly associated with affective ToM tasks (Mier et al., 2010). Similarly, Völlm et al. (2006) showed that affective ToM tasks were associated with activation of the paracingulate, ACC, PCC and the amygdala. Schlaffke et al. (2015) investigated overlapping and distinct brain regions for affective and cognitive ToM by using the same set of stimuli for all conditions, but using different questions that prompted either cognitive or affective ToM. Their results showed overlapping regions similar to those reported before in the mPFC and TPJ but also showed some important distinctions. Cognitive ToM recruited the precuneus, cuneus and temporal lobes, whereas affective ToM recruited areas in the basal ganglia, PCC and prefrontal cortices. In addition, they were able to achieve an 85% accuracy at distinguishing between the two ToM conditions using a multivariate pattern classifier.

Interestingly, there is a large overlap between the regions identified for the social brain and the Default Mode Network (DMN; Mars et al., 2012). The DMN is affected in ASD (Padmanabhan et al., 2017), which suggests that there may be an association between social cognition and the DMN. Additionally, this disruption may be related to dopamine, which has been shown to be associated with the function of the DMN (Spindler et al., 2021) and has been implicated in ASD (Pavăl, 2017). The neurotransmitters GABA and glutamate have also been implicated in ASD (Zhang et al., 2020; Dai et al., 2021). Serotonin has also been implicated in ToM abilities, specifically in moral judgements (Crockett et al., 2010, 2015; Kanen et al., 2021).

In summary, ToM clearly involves the mPFC and TPJ, but there are some specific brain areas that are specialized depending on the ToM task employed. This further highlights that ToM is a complex cognitive process that involves a number of smaller sub-processes. Understanding the structure of the neural networks that are involved in human ToM may provide insights into developing suitable models and architectures for machine ToM. An example of how knowledge of brain regions involved in ToM can inform the development of computational models, Zeng et al. (2020) propose a brain inspired model of belief ToM using high-level knowledge of the functions of different brain regions relevant for ToM and test it on two simple false belief tasks. Importantly, evidence from both cognitive and neuroscience research demonstrates that ToM is comprised of a number of functions that have specialized neural underpinnings, but there is a core network that seems to be active across a number of tasks. As such there should be a more holistic approach to machine ToM, although computational models with more specialized subfunctions may also be useful. The use of human neuroimaging data to train AI models is becoming increasingly popular (see Section Use of Human Data).

### Findings and Implications for ToM in AI

Given that ToM is key to understanding social contexts, it is increasingly important to ensure that AI with social applications have at least some ToM. For example, autonomous cars are required to interact with human drivers and pedestrians safely. If a child is on one side of the road and an ice cream truck on the other, there is a likelihood that the child will cross the road to get some ice cream. In this situation, a human driver would take the precaution to slow down and be vigilant of any sudden movement from the child, such as running across the road to the ice cream truck. Without understanding the possible intentions of the child, an autonomous car may not see this as a potential hazard. Similarly, in the healthcare setting, there is a drive toward empathetic healthcare, where robots can assist in the daily lives of patients by acknowledging their physical, mental and emotional needs in order to cater for them. More in general, ToM is essential for AI in human-robot interactions, such as for surgical robotics. This goes beyond simple emotion recognition, as all aspects of ToM are involved. This is where cognitive and neuroimaging studies may be useful to inform how best to create the appropriate AI models. While machine ToM can learn a lot from human ToM, even in more specific sub-functions, one of the main differences seems to be the lack of a holistic approach in AI. It is worth noting, that as AI becomes more readily used in society, there will be a need for feedback between AI and human agents, which would result in higher-order ToM requirements. However, given the lack of AI models with first-order ToM abilities, we have focused on this first-order ToM ability in the current review. Some examples of recent proposals of recursive reasoning models, relevant for higher-order ToM (in a Reinforcement Learning framework), include Wen et al. (2019) and Moreno et al. (2021).

## Computational and Preference ToM in Artificial Intelligence

A key conclusion from our review of ToM in cognitive and neurosciences is that learning, and in particular life-long learning, is a key component of ToM. In this second part of the survey, therefore, we will review ToM-related proposals within AI, with a focus on relatively recent proposals which include a learning component and which are not already covered extensively in other literature reviews/surveys. We also focus more on preference ToM (algorithms which focus on inferring preferences, rather than more generally on inferring mental states), as there has arguably been more recent work on preference-ToM than on belief-ToM (e.g., in the fields of Inverse Reinforcement Learning—IRL—and Preference Learning), and it has been shown that ToM can indeed be cast as an IRL problem (Jara-Ettinger, 2019). For broader surveys with less of a focus on very recent IRL and preference learning algorithms, see Rusch et al. (2020) and Gonzalez and Chang (2021). We also focus on first-order ToM models, since first-order ToM is already challenging enough for current AI models. See Gonzalez and Chang (2021) for a discussion about computational models of higher-order ToM and higher-order ToM in humans and Arslan et al. (2017b) for an example of a model of children's development of second-order ToM. Some examples of recent proposals of recursive reasoning models, relevant for higher-order ToM (in a Reinforcement Learning framework), include Wen et al. (2019) and Moreno et al. (2021). Finally, a significant portion of work in AI has focused on modeling humans, more generally, for various purposes. An extensive review is beyond the scope of this survey, but we point the interested reader toward the following brief surveys on student (Chrysafiadi and Virvou, 2013; D'mello and Graesser, 2013) and user modeling (Biswas, 2012) and toward a brief summary of recommender system surveys (Zhang, 2019), as example overviews of the extensive work in AI that has gone into modeling humans more broadly.

### Cognitive vs. Affective ToM in AI: A Bird's Eye View

We first briefly discuss the current state of the research in belief inference and emotion inference ToM algorithms, and point the interested reader to more in-depth surveys.

Gonzalez and Chang (2021) split computational models of ToM into several broad categories, these include Game ToM, Observational Reinforcement Learning, Inverse Reinforcement Learning and Bayesian ToM. We cover these concepts to varying degrees in this review, but detailed reviews for each are provided in the following papers (Game ToM in Yoshida et al., 2008; Observational Reinforcement Learning in Albrecht and Stone, 2018; Inverse Reinforcement Learning in Arora and Doshi, 2021 and Bayesian ToM in Baker et al., 2011). For a selection of computational models of ToM using cognitive architectures, see e.g., Trafton et al. (2013); Hiatt and Trafton (2015); Arslan et al. (2017b).

In Game ToM, players' beliefs are represented using probability distributions over actions, states, or other players' beliefs. Psychological Game Theory, a subcategory of Game ToM, models motivations that depend on beliefs (either one's own or another's) and has been used to build formal operationalisations of emotions, as well as to model how players perceive other players' intentions behind their actions [see Gonzalez and Chang (2021) for more details]. For more examples of multi-agent models of ToM (including Game ToM) see de Weerd et al. (2017, 2018); Veltman et al. (2019). Bayesian ToM (Baker et al., 2011) models both beliefs and rewards in a Partially Observed Markov Decision Processes (POMDP) setting, using Bayesian inference. Currently, though, we still have little mechanistic knowledge of how humans perform ToM inferences and the current modeling oversimplifies the mental operations and the environments, leading to uncertainty about how well the current models will generalize (Gonzalez and Chang, 2021). Rusch et al. (2020) propose a typology of ToM tasks and computational models across the two dimensions of interaction and uncertainty, suggesting that it is necessary to take into account relatively high levels of both uncertainty and interaction in order to effectively model the more sophisticated ToM processes.

The central role of hot cognition and emotions in ToM has been recognized by several authors. Some examples of early work relevant to affective ToM include work on modeling a user's emotions and engagement in educational games (Conati, 2002) and on recognizing the emotions of a user interacting with an educational computer game (Conati and Maclaren, 2009). Ong et al. (2019) survey previous work on emotion ToM - computational models of how observers understand others' emotional states. They point out that there has been less work on inferring others' emotional states (falling under the category of affective cognition) than on inferring their mental states and that many well-known ToM models (like Bayesian ToM), often neglect emotions. They derive a taxonomy for affective cognition inferences, in the framework of an intuitive theory of emotions, and formalize emotion as part of a graphical model (which also includes, among others, beliefs, desires and actions). The taxonomy includes and surveys existing models of: emotion recognition (how observers infer emotions from facial expressions and body language), third-person appraisal (how people reason about an agent's response to an experienced event), inferring causes of emotions, emotional cue integration (how to integrate and reconcile multiple cues to an agent's emotions), reverse appraisal (inferring beliefs and desires from emotions), prediction (of others' potential behavior given a future or hypothetical emotion) and counterfactual reasoning and explanations (reasoning about others' emotions in states of the world that are different from the existing reality). The proposed graphical model formalization allows for unifying the various types of reasoning from the taxonomy as Bayesian inference within a common “intuitive Theory of Emotion.” For some of the categories from the taxonomy no computational models are proposed, but the authors argue that such models can be built under their proposed “intuitive Theory of Emotion” framework, using graphical model tools. Ong et al. (2019) also discuss the main challenges ahead for models of affective cognition: finding suitable computational representations of emotions (instead of the binary or multinomial labels that computational models currently use) and moving toward more real-world settings, with their richness of naturalistic data and contexts, and away from laboratory ones.

A detailed discussion of the advantages and disadvantages of the different major computational approaches to ToM is outside the scope of this paper and at least partly covered by Gonzalez and Chang (2021), but we will summarize some high-level points here. Bayesian ToM (e.g., Baker et al., 2011) seems particularly well-suited to model the inherent uncertainty that comes with trying to infer unobservable mental states and has previously been found to capture well the judgments of experimental participants (Baker et al., 2011). However, Bayesian models' scalability is often problematic and the scenarios that Bayesian ToM has been tested in are often quite simplistic. Cognitive architecture models like Zeng et al. (2020) have the advantage of often trying to directly model specific brain regions, but they might be harder to motivate in a principled manner at the computational level. Game ToM models (Yoshida et al., 2008) offer the advantage of conceptual analysis using concepts like Nash equilibria [see Gonzalez and Chang (2021) for more details], but the game settings they are used in are often too simple. Finally, RL models (including IRL and MARL) can allow for state-of-the-art results on real-world tasks and can be highly scalable, but can also require large amounts of computation (as well as data, or access to a simulator) for training and be less interpretable.

The overall impression is that past and existing work in the area has so far failed to propose an integrated, holistic approach to ToM able to combine both cognitive and affective components.

### Fundamental Results on Preference ToM

In the Reinforcement Learning (RL) framework, intelligent agents take actions in an environment so as to maximize cumulative reward. While in RL the reward is usually specified and the agent has to infer the optimal policy, in Inverse Reinforcement Learning (IRL) the agent tries to extract a reward function from observed behavior. *Rewards* or *preferences* are indeed a component of ToM, as discussed above. Ng and Russell (2000), for instance, formulate IRL as a linear programming problem and make the assumption that the observed behavior comes from an optimal *policy* (decision strategy). As a concrete example, an autonomous vehicle (AV) could be trained using RL to maximize the ratings (which would correspond to the rewards in the RL framework) it obtains from its passengers. In this same case, IRL could be used to e.g., infer what rating (reward) a particular AV drive would have obtained. This could be achieved by, for example, training a system which predicts ratings from drives by using historical data pairs of (drives, ratings). This rating/reward predictor could then be used to further improve the AV's driving through RL.

#### The Unidentifiability Conundrum

One of the problems of this setup, though, is the unidentifiability of the reward function: there are many reward functions for which the observed policy is optimal. Clifton (2021) discusses the unidentifiability issue in multi-agent settings (using an example from the ultimatum game). The conclusion is that, in theory, it is impossible to uniquely recover a player's reward function from their actions only. In other words, the underlying motivations cannot be unambiguously extracted from behavior.

Mindermann and Armstrong (2018) have shown that it is impossible to infer the reward function of an agent with unknown rationality. This holds even when we have access to the full human policy (i.e., a description of how a particular human responds to all possible inputs), because there are infinitely many possible ways of decomposing any policy into a planning algorithm and a reward function. Penalizing more complex (planner, reward) pairs does not address this problem either. A second result shows that for any inferred (planner, reward) pair, we cannot rule out that maximizing the inferred reward leads to at least half of the worst-case regret [where *regret* is defined as the difference between the (discounted) cumulated reward of the optimal policy and the (discounted) cumulated reward of the actual policy] with respect to the true reward. The authors argue that the true (planner, reward) decomposition must be very complicated since it has to encode the nuances of systematic human biases in decision-making. Noting that humans seem able to make inferences about other humans' preferences as part of their ToM abilities, they propose that “normative assumptions” (“key assumptions about the reward function and/or planner, that cannot be deduced from observations”) will have to be built into the inference algorithm and that humans must be using such shared priors.

The theoretical impossibility of inferring a human's reward function without additional assumptions, even with access to large amounts of behavioral data (e.g., their full policy), is suggestive of the promise of extracting insights from the workings of the brain structure more directly involved in ToM in humans (e.g., the mPFC and TPJ, see Section Findings and Implications for ToM in AI), especially in terms of biases and priors, induced by either genetics or the environment, or for somehow learning these biases and priors from cognitive data. These can play the role of the above-mentioned “normative assumptions.”

#### Overcoming Unidentifiability

Other approaches have been proposed to address the issue. Abbeel and Ng (2004) weaken the assumption about the observed behavior (“expert demonstrations”) to near-optimality and can output a RL policy with performance [measured in (discounted) cumulative reward] close to that of the expert, without needing to infer the underlying reward function (thus bypassing the issue of reward unidentifiability), by using feature matching (between demonstrations from the learned policy and demonstrations from the expert). The assumption is that the reward function is a weighted linear combination of features of the state. Nevertheless, many policies can satisfy the feature matching condition. Ziebart et al. (2008) propose choosing the policy with the maximum entropy. Further, Ziebart et al. (2010) replace the maximum entropy in the previous algorithm with the maximum causal entropy, allowing for a more principled algorithm in which each action's entropy is only conditioned on the previous states, and not future states. Shah et al. (2019a) propose another way to potentially overcome the impossibility result from (Mindermann and Armstrong, 2018), by learning the cognitive biases of the demonstrator, which are assumed to be encoded in their planner, in the same framework where a policy can be decomposed into a (planner, reward) pair. We can then find the reward function that results in the observed policy and optimize for it using RL, resulting in a bias-free policy.

#### Tackling False Beliefs

A number of interesting papers, such as Dafoe et al. (2020), discuss the importance of understanding others in multi-agent settings and how false beliefs about others' beliefs and preferences can lead to defection and suboptimal results, even in settings where cooperation is optimal for all the agents. This is a difficult problem, because preferences might not be defined explicitly or might even be incoherent. Tackling false beliefs would mark a clear step forwards toward a more integrated approach to at least cognitive ToM, within the RL/IRL setting. Reddy et al. (2018) also tackle the problem of inferring both beliefs and preferences from human behavior, in particular those of an expert having a wrong model of (beliefs about) the environment, in the IRL setup (in contrast, most IRL algorithms assume that the human expert has an approximately optimal model of the environment). It proposes to learn the expert's model by assuming access to multiple tasks with known reward functions, which helps overcome the (unidentifiability) problem that many different such models could be compatible with the observed data from a single task. The reward function for a new task can then be inferred using maximum causal entropy IRL.

#### Key Messages

Prior art in ToM for preference learning in AI has mostly been conducted within the mathematical framework of (inverse) reinforcement learning, with key results focusing around the impossibility or inferring even a simplified mind model of agents (purely based on rewards or preferences) in the absence of additional assumptions on the problem. As there is strong evidence humans are capable of ToM (albeit in a somewhat imperfect form), this strongly points at the need for further work on what these assumptions and priors should be. Computational ToM has also mainly focused on single aspects of the problem, with an apparent lack of holistic approaches. Neuroscientific evidence can arguably provide useful insights on both issues.

### Source of Information on Preferences

Human preferences can be learned from different information sources, such as expert demonstrations, comparisons of preferred trajectories from expert demonstrations, or proxy rewards - rewards provided by programmers which capture imperfectly some aspects of human preferences. The latter can be good specifications for scenarios from the training distribution, but not necessarily for novel situations which might occur during deployment [see Hong et al. (2020) for more examples]. Different sources come with various tradeoffs attached, e.g., in terms of how much information about preferences they actually provide and how costly gathering this information is.

A few papers deal with these issues. Hong et al. (2020) provide a simple formalism which can unify previously-proposed algorithms for learning preferences from different information sources in a Bayesian setting, by inferring a distribution over the possible rewards. Their framework can also integrate more exotic types of feedback, such as human decisions whether to turn an AI off, credit assignment (where the subset of the trajectory that has maximal reward is provided) and meta-choice (how the choice over what feedback to offer can itself provide information about the reward function). The key idea in Shah et al. (2019b) is that the state of the world has already been optimized for human preferences, so we can infer them in a RL framework just by looking at the world (e.g., observing a fragile vase intact on a table suggests humans probably care about it). Lindner et al. (2021) scale up this proposal using deep learning. This strand of research suggests that machines can make inferences on human mental states by observing the environment humans create around themselves, rather than human behavior alone (which remains crucial, see below).

More broadly, the question of what stimuli/inputs are relevant in the perspective of a machine ToM is a crucial one.

### Cooperation in Preference ToM

One of the most important paradigm shifts proposed in recent years with respect to preference ToM algorithms is the importance of the interaction between a single AI and its human overseer, in a cooperative setting, conceptualized in the framework of assistance games, which we detail below. This is in line with another conclusion from Section Cognitive and Neural Mechanisms of Theory of Mind, that ToM development is a social process which is facilitated by more frequent interactions with other people.

#### Beneficial Machines

Rather than coding machines in order for them to optimize a certain objective (which, if misspecified, can lead to failure), Russell (2019) proposes that we aim for machines to be *beneficial* to humans: “Machines are beneficial to the extent that their actions can be expected to achieve our objectives,” and should be designed according to the following principles:

1. “The machine's only objective is to maximize the realization of human preferences.”
2. “The machine is initially uncertain about what those preferences are.”
3. “The ultimate source of information about human preferences is human behavior.”

In a way, achieving “beneficial” machines is the whole purpose of endowing AI with ToM capabilities. However, it is important to note that in real-life contexts, human behavior is often constrained by moral, ethical, social and financial considerations.

#### Assistance Games

Hadfield-Menell et al. (2016) provide a formalization of the three principles above, in a setting including a human *H* and a robot *R* which tries to maximize *H*'s objective (corresponding to principle 1 above), which only *H* knows, and for which *R* only has a probability distribution (principle 2). *R* assumes that *H* chooses actions optimally according to their reward (corresponding to principle 3). In this *assistance game, H*'s best strategy is to teach *R* about the reward and *R*'s is to interactively learn and act. Ho et al. (2016) have shown that humans tend to be pedagogic when teaching, picking trajectories that help disambiguate their preferences, rather than optimal ones for the task.

Such explicit teaching frameworks recall efforts to “train” ToM to humans using demonstrations (Section Assessing Theory of Mind). In this assistance games framework, humans are modeled as part of the environment and their preferences are expressed *via* a latent variable that the AI can infer. In the reward learning framework considered in the first part of Section Computational and Preference ToM in Artificial Intelligence, instead, the AI learns a reward model from human feedback which is external to the environment. Shah et al. (2021a) compare the two approaches and claim that, by merging reward learning and control in a single policy, assistive agents can reason about the impact of control actions (such as asking questions) on the reward learning. This allows them to choose what questions to ask the human based on their relevance, to create plans whose success depends on future feedback and to learn not just from explicit communication, but also from physical human actions. The “asking questions” behavior is not hardcoded, but emerges from the interaction between the human and the AI. This is in line with what we observed in Section Development of Theory of Mind, about the paramount importance of language in ToM development in humans. Clearly human behavior (principle 3 above) is meant to include linguistic interactions as well.

Woodward et al. (2020) combine deep RL with assistance games, in that a principal and an agent have to pick fruit in a gridworld but only the principal knows which fruit is rewarding. They show that the agents can learn to cooperate simply as a result of joint training, without needing explicit demonstrations or trajectory comparisons. Examples of emergent behaviors are a restricted field-of-view agent learning to follow the principal to see which fruit it prefers and the agent learning to communicate its uncertainty about the preferred fruit, while the principal learns to “answer” through its movements. They also show that human/AI pairs obtain better performance than single humans on this task, reinforcing our conclusions in Section 2.5 about the importance of cooperative settings and the opportunity of exploring ideas from developmental robotics.

#### Evolving Preferences

While standard IRL algorithms assume that humans' preferences are fixed, Chan et al. (2019) model humans as learning their preferences, using an assistive *multi-armed bandit* setting (Slivkins, 2019) in which humans repeatedly choose one of several arms of different slot machines with unknown reward distributions to pull. They are aided by a robot which can intercept the player and pull an arm of its own choice and can only see the human's arm pull choices (not the rewards). This formalizes the setting of an AI with partial information trying to help a human who is learning their preferences. Using both a theoretical analysis and an experimental setting, the authors find that better human performance in isolation does not necessarily lead to better performance of the human-robot team (since information can also be communicated through the arm pulls) and that robots which model humans' learning tend to do better, even when the model is wrong. The problem, however, is very sensitive to the human's learning model and the robot's assumption about it. More generally, human goals evolve over time, so an effective computational ToM approach arguably needs to model mental states in a dynamic, rather than static way.

### Modeling Preference Uncertainty

ToM can be seen as a process in which unobservable, latent variables (e.g., goals, preferences, emotional states, and intentions) need to be inferred from the available observable quantities (human behavior and scene context). As such, ToM is inherently subject to severe uncertainty, as mental states are by definition inaccessible.

Consequently, incorporating measures of uncertainty about the learned preferences and trying to handle misspecification of the space of possible reward functions have received increasing attention in preference ToM lately. As a proof of the importance of assessing uncertainty, Hadfield-Menell et al. (2017a) use the assistance game framework to study how the robot *R*'s uncertainty (about the human *H*'s preferences) impacts the incentives around an off-switch, which *H* can use to switch *R* off. It showed that, generally, more uncertainty on *R*'s side leads to more deference toward *H* (allowing *H* to shut off *R*), though at the cost of *R* being less able to help *H* when it is very uncertain about the reward.

The mainstream approach to modeling uncertainty in (inverse) reinforcement learning is Bayesian IRL. Ramachandran and Amir (2007) propose using a distribution over the inferred reward function (as opposed to non-Bayesian IRL, which produces a point estimate), which can be used to plan conservatively (taking into account the worst potential outcomes), also known as risk-averse planning, with potential safety benefits. Bayesian IRL algorithms are typically computationally expensive, though. Brown et al. (2020) propose a much faster algorithm, based on learning from preferences over demonstrations. The inferred uncertainty over the reward allows for confidence intervals around the performance of the policy to be estimated and makes the reward model more robust to reward hacking (which can be detected as high variance in the reward model's estimate).

Hadfield-Menell et al. (2017b) propose reinterpreting the common hand-coded reward functions in RL as only providing information about the AI's optimal behavior in the training environment (rather than more generally), using a *Boltzmann rationality model*. The latter assumes that a hand-coded reward is more likely to be picked by the programmer (from the space of possible hand-coded rewards) if it leads to higher true reward in the training environment. We can then perform Bayesian inference to obtain a probability distribution over the true reward function, which can be combined with risk-averse planning to avoid negative side effects that the AI has never encountered before. In a slightly different approach, Bobu et al. (2018) propose one algorithm to check whether the human's true reward function is outside the robot's hypothesis space on a task using learning from physical human corrections, by checking whether all corrections appear irrelevant to the robot. Jonnavittula and Losey (2021), instead, propose to mitigate the effects of misspecification by making sure we underestimate the demonstrator's capabilities (how good are the demonstrations they can provide).

Overall, the state of the art so far seems to neglect the modeling of uncertainty of other aspects of ToM other than goals/rewards/preferences, with the partial exception of Bayesian ToM (see Section Cognitive vs. Affective ToM in AI: A Bird's Eye View). A proper accounting of the severe uncertainties involved is likely to be key for future work in this area. Additionally, existing efforts focus on Bayesian inference, which models uncertainty using classical probability theory. Methods from evidential (Sensoy et al., 2018) or epistemic (Cuzzolin, 2021) artificial intelligence, which model “second order” uncertainty about the probabilities themselves may be interesting to consider to provide a safe and robust approach to ToM in AI.

### Multi-Task Learning, Meta-Learning, and Continual Learning

Learning is a key component of ToM. However, when naively implemented this can be prohibitively expensive, given the level of challenge involved. In particular, learning a specification using preference ToM algorithms can often require large amounts of data. In situations in which we have access to data from multiple overlapping tasks, however, we can employ techniques from multi-task learning (Caruana, 1997), meta-learning (Vanschoren, 2018), and/or continual learning (Van de Ven and Tolias, 2019) to learn from the available training data more efficiently.

Multi-task learning, meta-learning and continual learning are all motivated by the idea of reducing the amount of data required for machine learning algorithms to learn different tasks, by leveraging the shared structure of multiple related tasks. The differences between these three types of algorithms are as follows. In multi-task learning, the data from all the different training tasks is usually all available from the beginning of the training phase and the goal is to try to solve all the training tasks. In meta-learning, instead, the goal is to use the training tasks in order to solve new (test) tasks from a small amount of data. Finally, in continual learning, the goal is to learn a model for a large number of tasks sequentially without forgetting knowledge obtained from the preceding tasks, where the data in the old tasks is not available any more during training new ones.

#### Meta-Learning

One prototypical meta-learning algorithm is Model-Agnostic Meta-Learning (MAML; Finn et al., 2017), which explicitly trains the parameters of a model so that a small number of gradient steps with a small amount of training data from a new task will produce good generalization performance on that task. Xu et al. (2019) adapts MAML to maximum entropy IRL (Ziebart et al., 2008) by learning a “prior” over reward functions which is specifically optimized so that the reward function corresponding to a new task can be learned from a limited number of demonstrations. Rabinowitz et al. (2018) shows a proof of concept of machine prediction of other agents' false beliefs, inspired by the cognitive Sally-Anne test in humans, by formulating ToM as a meta-learning problem. There, an observer meta-learning agent (called ToMnet) parses the episodes of many agents in many simple gridworld environments, so as to learn a prior over the behavior of an agent type. At test time, ToMnet can infer the type of a novel agent and use recent episodes of experience of that agent, as well as its trajectory on the current episode, to predict the agent's future behavior. It is also shown to be able to perform few-shot IRL, by inferring the goals of agents (state-based reward functions defined over simple gridworlds). When trained on deep RL agents, ToMnet implicitly learns that other agents can hold false beliefs (about these simple gridworlds) and it can also be trained to predict agents' belief states (which helps with revealing them). It can then infer these beliefs from behavior alone. The authors interpret their results as demonstrating “that representational Theory of Mind” can arise simply by observing competent agents.

#### Multi-Task Learning

An example of multi-task IRL is (Dimitrakakis and Rothkopf, 2011), which extends Bayesian IRL (Ramachandran and Amir, 2007) to the multi-task setting by using multiple structured priors which capture the relatedness of different tasks on reward functions and policies. The authors show that this allows them to both learn efficiently from multiple experts and differentiate between their goals, in experiments on simple MDPs.

#### Continual Learning

Mendez et al. (2018) propose a continual IRL algorithm in the maximum entropy IRL framework (Ziebart et al., 2008), by assuming that the structure of the multiple tasks is shared and by using a sparsity reward prior over the reward functions corresponding to different tasks. The authors show that they can obtain better performance than the baseline single-task maximum entropy IRL algorithm (which learns separate reward functions for each task, without any shared structure), with little computational overhead.

#### Key Messages

These efforts seem to confirm that ToM can develop more efficiently, and by using much less data, in a life-long learning scenario in which machines can observe humans perform a variety of tasks. Results also seem to indicate that ToM in machines (and possibly in humans) may be considered a form of “emergent” behavior.

Meta-learning also appears to be, in our view, a critical component of a fully-fledged machine ToM, as the task consists in learning how different agents (e.g., different individual humans) behave as a function of their rich mental states.

Continual learning *per se* seems to have received less consideration so far in ToM efforts in AI (albeit one might argue that RL is an intrinsically sequential/continual learning setting). One reason might be that the continual learning community has so far mostly focused on supervised settings, whereas, as we argued above, any efficient ToM learning mechanism must be able to leverage a small number of labeled examples.

### Benchmarking and Evaluation

One of the difficulties involved in building better ToM algorithms, especially in the case of preference ToM, concerns the evaluation of such algorithms. Many of the tasks we would want to be able to solve do not possess specifications which can easily be captured using hand-crafted code. More generally, human preferences are vague and hard to specify. This suggests that a good benchmark of preference inference algorithms should probably have humans evaluate the performance of these algorithms, because, if we had a signal that allowed us to automatically evaluate such algorithms, that signal could also be used for learning (Shah et al., 2021b).

Shah et al. (2021b) introduce the MineRL BASALT competition, with tasks aiming to be realistic, in the sense of it being challenging to write reward functions for them and there being many other potential goals in the environment than the one intended. The Minecraft game was chosen for these reasons, with tasks such as “create a waterfall and take a scenic picture of it.” The chosen tasks are inherently vague and hard to formalize and the agents are evaluated by humans. Inspired by false-belief ToM evaluation protocols in cognitive science, Nematzadeh et al. (2018) and Le et al. (2019) have proposed first-order and second-order belief evaluation tasks for language models (also see Section Dysfunction of Theory of Mind for discussion about the interaction of ToM and linguistic abilities in humans). Sap et al. (2019) have proposed a dataset to evaluate language-based commonsense reasoning about social interactions, including reasoning about motivation (preference ToM—relevant) and about emotional reactions (relevant for affective ToM). Zellers et al. (2021) introduce an evaluation method for NLP (natural language processing) models for tasks in which there is no literally correct answer. In TuringAdvice (Zellers et al., 2021), an NLP model must provide a helpful response in a situation where a human is asking for advice, with model responses compared against good human responses and the response considered successful if it is at least as helpful to the advice-seeker as the human-written one. The authors also show that there is still a large gap between the best language models and human-written advice on this task.

Bard et al. (2020) propose the cooperative, imperfect information card game Hanabi as a challenge benchmark, since it requires reasoning about the beliefs and the intentions of other players, focusing on the *ad-hoc* setting where an agent has to coordinate with a team they encounter for the first time. Choudhury et al. (2019) compare a preference ToM-based learning algorithm with two other non-ToM algorithms on a task where an autonomous vehicle (AV) interacts with a human-driven one. The preference ToM-based approach models the human as approximately optimizing an unknown reward function, to then use planning to determine the AV's actions. The second algorithm, called Black-box model-based learning, also uses planning, but trains a neural network to directly predict human actions. The third algorithm, model-free learning, just uses a deep RL algorithm to directly output the AV's actions. They find that the ToM-based method is much more sample-efficient and more robust to changes in the domain distribution when not much data is available, but with enough data the second algorithm dominates. If the preference ToM assumptions are significantly violated (which is quite likely in practice, because of misspecification), then the black-box model-based algorithm will vastly outperform. In their setup, the model-free algorithm did not work at all.

Clearly, the establishment of commonly accepted criteria for the design and implementation of ToM benchmarks and the associated evaluation protocols is still in its infancy, as arguably a serious stumbling block in the further development of the field.

## Cognitive vs. Computational ToM: a Discussion

As evidenced by the neuroimaging of ToM (Section Cognitive and Neural Mechanisms of Theory of Mind), there appear to exist specialized, context-dependent applications of ToM, but also a core structure to human ToM that is present in any task requiring ToM abilities. As illustrated above, AI has largely focused on single aspects of the problem, rather than propose more holistic approaches to computational models of ToM. This may be largely due to the fact that the notion is difficult to conceptualize mathematically, but also computationally expensive. Much, however, can be learned from cognitive psychology and neuroscience for the purpose of developing computational models with ToM.

A number of potentially useful insights and conclusions have been already drawn throughout our exposition of Sections Cognitive and Neural Mechanisms of Theory of Mind and Computational and Preference ToM in Artificial Intelligence. We wish to conclude the paper with some additional critical reflections.

### Current State of ToM in AI

Our review of the prior art in Section Computational and Preference ToM in Artificial Intelligence shows that there has been significant progress on the theoretical side, especially in preference ToM, e.g., combining different information sources about human preferences (Hong et al., 2020) and integrating uncertainty (Hadfield-Menell et al., 2017b) and human interaction (Shah et al., 2021a). On the other hand, some theoretical results (Mindermann and Armstrong, 2018) cast doubt on the possibility of completely inferring human preferences from observed behavior only, even with access to infinite amounts of behavior data. Using data and insights from cognitive sciences might be a way to bypass these impossibility results, since we know that humans can infer others' preferences satisfactorily. A related limitation of current preference ToM algorithms is the large amounts of data they require in practice to learn ToM capabilities which are still inferior to humans; Jara-Ettinger (2019) points out that the machine ToM experiments reported in Rabinowitz et al. (2018) required 32 million samples to learn to perform goal inference at a level similar to that of a 6-month-old infant. If infants learned ToM in this way, 175,000 labeled demonstrations would be required every day during those 6 months. Cognitive sciences might help build machine ToM algorithms which need less data.

### Use of Human Data

One possibility is to use human derived data, be it brain or behavioral data, to develop AI models. There are already some cases in the literature of brain data being used to improve computational models. Kim et al. (2017), for instance, use error-related potentials from EEG signals, for implicit feedback, to improve gesture-based robot control during human robot interactions. A recent review provides details as to how brain computer interfaces and neurofeedback research is now being used to estimate cognitive load, attentional level, perceived errors and emotions from brain signals to improve interactions between humans and robots (Alimardani and Hiraki, 2020). Zeng et al. (2020) propose a brain inspired model of belief ToM using high-level knowledge of the functions of different brain regions relevant for ToM and test it on two simple false belief tasks. It could then be possible to use human data from ToM tasks to develop AI models with better ToM abilities. For example collecting data from human drivers on reactions in various situations and using this data to train autonomous cars. However, such an approach also has limitations. To develop accurate AI models large amounts of data are required, collecting human data is often expensive (especially in the case of neuroimaging data). There are now projects such as the Human Brain Project (HBP; https://www.humanbrainproject.eu/en/) and UK Biobank (https://www.ukbiobank.ac.uk/) that are actively collecting neuroimaging measures from thousands of participants; unfortunately, these measures are not directly related to ToM. As stated above, the study of the structures more directly involved in ToM in humans and their associated processes could inform new architectures and classes of computational models specifically suited to ToM.

### Ethical Issues

It is not within the scope of this review to fully review the ethical concerns of ToM research in AI; nevertheless, we feel obliged to provide a brief overview here. Obvious ethical concerns exist when handling human data (anonymity and privacy). With the movement toward open science and data sharingthis might become less of a potential limitation when it comes to historical data. When considering the improvement of AI models, particularly those that may have ToM abilities, several ethical considerations need to be taken into account. Better machine ToM technology could increase concerns about machines violating the privacy of others' minds, though (e.g., at deployment). We also discuss additional safety-relevant concerns in section Safety Concerns. Dafoe (2018) states a number of risks with increasing AI technology including labor displacement, inequality, strategic instability, and an AI race that sacrifices safety and other values. As yet there seems to be relatively little policy guidance governance regarding AI (Dafoe, 2018). Take autonomous driving cars. When an accident occurs, who should be considered at fault: the driver, the company who manufactured the car, the programmer who developed the AI system in use? Similarly, if we develop AI systems or robots that are able to replace human workers, what happens economically to the displaced workforce? There are organizations, e.g., the Centre for the Governance of AI (GovAI: https://governance.ai/) that attempt to maximize the benefits, whilst managing the risks, of artificial intelligence. GovAI research is used to advise decision-makers in private industry, civil society, and government. An additional ethical concern relates more to the field of machine ethics. Moor (2011) argues that there are four types of machine agents: ethical impact agents; implicit ethical agents; explicit ethical agents and full ethical agents. An AI endowed with ToM abilities would be considered a full ethical agent, but this may suggest that the AI should have moral patiency and as such deserving of moral consideration. Harris and Anthis (2021) survey the literature on the moral considerations of artificial entities.

### A Contrarian View

It might also be the case that no further cognitive science inspiration or data is necessary for advanced ToM capabilities. One plausible scenario is for the currently observed growth in capabilities of language models to continue, as computing power is scaled up (Kaplan et al., 2020), potentially all the way to human levels of performance (Branwen, 2021). A number of people argue that a language model with human-level prediction performance would have to have acquired advanced (implicit) ToM capabilities (as it is hard to imagine human-level language modeling otherwise). While the required amounts of computational resources to accomplish this might be beyond what is available today, it is not clear that they will still be unavailable within a few decades, given continuously decreasing costs, algorithmic progress and increased willingness to spend [see Karnofsky (2021)]. As a result, this might end up being the most direct path to more powerful AI (Sutton, 2019), including ToM abilities.

### Safety Concerns

Better ToM algorithms could also come with more safety concerns, especially if the improvements only concern the cognitive aspects of ToM, and not the affective ones too. An AI system with human models with a catastrophic bug might optimize for human suffering, and AI systems with human models might produce subsystems that are agent-like and thus dangerous, given that humans are agent-like (Kumar and Garrabrant, 2019). AI systems with better ToM capabilities could also be better at deceiving and manipulating people. In this context, one ToM-related problem which (in our view) appears to have received too little attention is how to have AIs be motivated to only try to fulfill human preferences, known as the problem of *intent alignment* (between an AI A and its human overseer H; Christiano, 2018). This is separate from the problem of having the AI be capable of inferring and fulfilling human preferences. One reason to focus more on this problem could be that we might expect AI capabilities of inferring and being able to satisfy human preferences to keep increasing, even without major conceptual breakthroughs (see previous paragraph), while having the AI be *intent aligned* seems like a separate problem, which increasing capabilities wouldn't solve by default. We can also draw some loose analogies from cognitive sciences, where some people can understand others' preferences (and, more generally, mental states) without being motivated by them (e.g., psychopaths); this provides a proof of existence of intelligences which are capable of inferring (some) human preferences without being motivated to follow them. Research from cognitive science on phenomena like psychological altruism, empathy (de Waal, 2008) and empathic concern (FeldmanHall et al., 2015) might help with clarifying this problem and might even provide inspiration for designing more *intent aligned* AI. Intent alignment alone might not be sufficient though. Additional risks (related to the governance of AI, also see Section Ethical Issues) include misuse (Brundage et al., 2018) and structural risks (e.g., altering global market structures, shifting military power, or undermining nuclear stability; Dafoe, 2020). The question of who the AI is aligned to is also important. Recent work has proposed a multi-principal (user) assistance game framework where an AI acts on behalf of N humans who may have very different payoffs (Fickinger et al., 2020).

## Conclusion

While it is clear that there has been progress on AI models for specific aspects of ToM, at least in limited settings, there needs to be a more holistic approach. Advancement in this field could be enhanced through interdisciplinary research, including psychologists, neuroscientists and those in mathematics and computing who have a special interest in AI. For example, this is being done as part of the HBP, where supercomputers are being used for AI simulation of the human brain (EBRAINS and SpiNNaker, https://www.humanbrainproject.eu/en/). It is recognized that part of the difficulty is that on a psychological level there is little evidence that the multiple components assessed by various tasks of ToM are indeed measuring the same construct. Factor analytical or principal component techniques using measures from a wide variety of tasks, such as those mentioned above, might be able to better elucidate this. However, there does seem to be some shared, core ToM network evidenced by neuroimaging, where a core network including the TPJ and mPFC is activated across multiple ToM tasks. In addition, the more direct use of human data in AI computation has been so far underexplored. This approach might lead to outperform current AI techniques, while allowing us to better benchmark the capabilities of machine ToM. Nevertheless, there have been great advances in the field, which holds promise for eventually being able to produce AI models which incorporate ToM for better use in society including healthcare and other industries.

## Author Contributions

All authors were involved in the conceptualization, writing, and reviewing of this manuscript. All authors contributed to the article and approved the submitted version.

## Funding

This work was funded by a Leverhulme Trust grant to FC and BS under the Research Programme Grant (RPG-2019-243). CL and BC were funded by the Leverhulme Trust (RPG-2019-243). BS and CL research work is conducted within the framework of the NIHR MedTech and *in vitro* diagnostic Co-operative (MIC) and the NIHR Cambridge Biomedical Research Centre (BRC) Mental Health and Neurodegeneration themes. This work was supported in part by the European Union's Horizon 2020 Research and Innovation Programme under Grant 964505 (E-pi).

## Conflict of Interest

FC is the Director of Oxford AI Consulting, and does consultancy for Huawei Canada. BS consults for Cambridge Cognition and Greenfield BioVentures. The remaining authors declare that the research was conducted in the absence of any commercial or financial relationships that could be construed as a potential conflict of interest.

## Publisher's Note

All claims expressed in this article are solely those of the authors and do not necessarily represent those of their affiliated organizations, or those of the publisher, the editors and the reviewers. Any product that may be evaluated in this article, or claim that may be made by its manufacturer, is not guaranteed or endorsed by the publisher.

## References

[B1] Abbeel P. Ng A. Y. (2004). Apprenticeship learning *via* inverse reinforcement learning, in Proceedings of the Twenty-First International Conference on Machine Learning (Banff Alberta), 1. 10.1145/1015330.1015430

[B2] Academy of Medical Sciences (2019). The Developing Brain in Health and Disease. Available online at: https://acmedsci.ac.uk/policy/policy-projects/the-developing-brain-in-health-and-disease (accessed September 9, 2021).

[B3] Albrecht S. V. Stone P. (2018). Autonomous agents modelling other agents: a comprehensive survey and open problems. Artif. Intelligen. 258, 66–95. 10.1016/j.artint.2018.01.002

[B4] Algarni M. (2017). Understanding the game theory of the prisoner's dilemma. Int. J. Appl. Inform. Syst. 11, 1–5. 10.5120/ijais2017451640

[B5] Alimardani M. Hiraki K. (2020). Passive brain-computer interfaces for enhanced human-robot interaction. Front. Robot. AI. 7:125. 10.3389/frobt.2020.00125 33501291 PMC7805996

[B6] Amodio D. M. Frith C. D. (2006). Meeting of minds: the medial frontal cortex and social cognition. Nat. Rev. Neurosci. 7, 268–277. 10.1038/nrn1884 16552413

[B7] Arora S. Doshi P. (2021). A survey of inverse reinforcement learning: challenges, methods and progress. Artif. Intelligen. 2021, 103500. 10.1016/j.artint.2021.103500

[B8] Arslan B. Hohenberger A. Verbrugge R. (2017a). Syntactic recursion facilitates and working memory predicts recursive theory of mind. PLoS ONE 12:e0169510. 10.1371/journal.pone.0169510 28072823 PMC5225003

[B9] Arslan B. Taatgen N. A. Verbrugge R. (2017b). Five-year-olds' systematic errors in second-order false belief tasks are due to first-order theory of mind strategy selection: a computational modeling study. Front. Psychol. 8, 275. 10.3389/fpsyg.2017.00275 28293206 PMC5329038

[B10] Auyeung B. Baron-Cohen S. Ashwin E. Knickmeyer R. Taylor K. Hackett G. (2009). Fetal testosterone and autistic traits. Br J Psychol. 100, 1–22. 10.1348/000712608X311731 18547459

[B11] Baker C. Saxe R. Tenenbaum J. (2011). Bayesian theory of mind: modeling joint belief-desire attribution, in Proceedings of the Annual Meeting of the Cognitive Science Society (Boston), 33.

[B12] Bard N. Foerster J. N. Chandar S. Burch N. Lanctot M. Song H. F.. (2020). The hanabi challenge: a new frontier for ai research. Artif. Intelligen. 280, 103216. 10.1016/j.artint.2019.103216

[B13] Barnes-Holmes Y. McHugh L. Barnes-Holmes D. (2004). Perspective-taking and Theory of Mind: a relational frame account. Behav. Analyst Today. 5, 15. 10.1037/h0100133

[B14] Baron-Cohen S. (1989). Are autistic children “behaviorists”? An examination of their mental-physical and appearance-reality distinctions. J. Autism Dev. Disord. 19, 579–600. 10.1007/BF02212859 2606886

[B15] Baron-Cohen S. (1995). Mindblindness: An Essay on Autism and Theory of Mind. Cambridge, MA: MIT Press. 10.7551/mitpress/4635.001.0001

[B16] Baron-Cohen S. (2000). Theory of mind and autism: a fifteen year review. Understand. Other Minds. 2, 102. Available online at: https://docs.autismresearchcentre.com/papers/2000_SBC_Theory-of-mind-an-autism-review.pdf

[B17] Baron-Cohen S. Goodhart F. (1994). The ‘seeing-leads-to-knowing' deficit in autism: the Pratt and Bryant probe. Br. J. Dev. Psychol. 12, 397–401. 10.1111/j.2044-835X.1994.tb00642.x

[B18] Baron-Cohen S. Leslie A. M. Frith U. (1985). Does the autistic child have a “theory of mind”? Cognition 21, 37–46. 10.1016/0010-0277(85)90022-8 2934210

[B19] Baron-Cohen S. Leslie A. M. Frith U. (1986). Mechanical, behavioural and intentional understanding of picture stories in autistic children. Br. J. Dev. Psychol. 4, 113–125. 10.1111/j.2044-835X.1986.tb01003.x

[B20] Biswas P. (2012). A brief survey on user modelling in human computer interaction, in Speech, Image, and Language Processing for Human Computer Interaction: Multi-Modal Advancement, ed U. S. Tiwary (Pennsylvania, PA: IGI Global), 1–19. 10.4018/978-1-4666-0954-9.ch001

[B21] Bland A. R. Roiser J. P. Mehta M. A. Schei T. Boland H. Campbell-Meiklejohn D. K.. (2016). EMOTICOM: a neuropsychological test battery to evaluate emotion, motivation, impulsivity, and social cognition. Front. Behav. Neurosci. 10, 25. 10.3389/fnbeh.2016.00025 26941628 PMC4764711

[B22] Bobu A. Bajcsy A. Fisac J. F. Dragan A. D. (2018). Learning under misspecified objective spaces, in Conference on Robot Learning PMLR (Zürich), 796–805.

[B23] Branwen G. (2021). The Scaling Hypothesis. Available online at: https://www.gwern.net/scaling-hypothesis (accessed September 1, 2021).

[B24] Brothers L. (1990). The social brain: a project for integrating primate behaviour and neurophysiology in a new domain. Concepts Neurosci. 1, 27–51.

[B25] Brown D. Coleman R. Srinivasan R. Niekum S. (2020). Safe imitation learning *via* fast bayesian reward inference from preferences, in International Conference on Machine Learning PMLR, 1165–1177.

[B26] Brundage M. Avin S. Clark J. Toner H. Eckersley P. Garfinkel B. (2018). The malicious use of artificial intelligence: forecasting, prevention, and mitigation. arxiv:1802.07228. Available online at: https://arxiv.org/ftp/arxiv/papers/1802/1802.07228.pdf

[B27] Brüne M. (2005). Theory of mind in schizophrenia: a review of the literature. Schizophr. Bullet. 31, 21–42. 10.1093/schbul/sbi002 15888423

[B28] Carpendale J. I. Lewis C. (2004). Constructing an understanding of mind: the development of children's social understanding within social interaction. Behav. Brain Sci. 27, 79–96. 10.1017/S0140525X04000032 15481944

[B29] Caruana R. (1997). Multitask learning. Machine Learn. 28, 41–75. 10.1023/A:1007379606734

[B30] Castelli F. Frith C. Happé F. Frith U. (2002). Autism, Asperger syndrome and brain mechanisms for the attribution of mental states to animated shapes. Brain 125, 1839–1849. 10.1093/brain/awf189 12135974

[B31] Chan L. Hadfield-Menell D. Srinivasa S. Dragan A. (2019). The assistive multi-armed bandit, in HRI '19: Proceedings of the 14th ACM/IEEE International Conference on Human-Robot Interaction (Daegu: IEEE), 354–363. 10.1109/HRI.2019.8673234

[B32] Choudhury R. Swamy G. Hadfield-Menell D. Dragan A. (2019). On the utility of model learning in HRI, in Proceedings of the 14th ACM/IEEE International Conference on Human-Robot Interaction (HRI '19). Piscataway, NJ: IEEE Press, 317–325. 10.1109/HRI.2019.8673256

[B33] Christiano P. (2018). Clarifying “AI Alignment”. Available online at: https://www.alignmentforum.org/posts/ZeE7EKHTFMBs8eMxn/clarifying-ai-alignment (accessed September 1, 2021).

[B34] Chrysafiadi K. Virvou M. (2013). Student modeling approaches: a literature review for the last decade. Expert Syst. Appl. 40, 4715–4729. 10.1016/j.eswa.2013.02.007

[B35] Clifton J. (2021). Weak Identifiability and Its Consequences in Strategic Settings. Available online at: https://longtermrisk.org/weak-identifiability-and-its-consequences-in-strategic-settings/ (accessed September 1, 2021).

[B36] Conati C. (2002). Probabilistic assessment of user's emotions in educational games. Appl. Artif. Intelligen. 16, 555–575. 10.1080/08839510290030390

[B37] Conati C. Maclaren H. (2009). Empirically building and evaluating a probabilistic model of user affect. User Model. User-Adapt. Interact. 19, 267–303. 10.1007/s11257-009-9062-8

[B38] Crockett M. J. Clark L. Hauser M. D. Robbins T. W. (2010). Serotonin selectively influences moral judgment and behavior through effects on harm aversion. Proc. Natl. Acad. Sci. U. S. A. 107, 17433–17438. 10.1073/pnas.1009396107 20876101 PMC2951447

[B39] Crockett M. J. Siegel J. Z. Kurth-Nelson Z. Ousdal O. T. Story G. Frieband C.. (2015). Dissociable effects of serotonin and dopamine on the valuation of harm in moral decision making. Curr. Biol. 25, 1852–1859. 10.1016/j.cub.2015.05.021 26144968 PMC4518463

[B40] Cuzzolin F. (2021). The Geometry of Uncertainty – The Geometry of Imprecise Probabilities. Cham: Springer International Publishing. 10.1007/978-3-030-63153-6

[B41] Cuzzolin F. Morelli A. Cirstea B. Sahakian B. J. (2020). Knowing me, knowing you: theory of mind in AI. Psychol. Med. 50, 1057–1061. 10.1017/S0033291720000835 32375908 PMC7253617

[B42] Dafoe A. (2018). AI Governance: A Research Agenda. Oxford: Governance of AI Program, Future of Humanity Institute, University of Oxford, 1442, 1443.

[B43] Dafoe A. (2020). AI Governance: Opportunity and Theory of Impact. Available online at: https://forum.effectivealtruism.org/posts/42reWndoTEhFqu6T8/ai-governance-opportunity-and-theory-of-impac (accessed February 22, 2022).

[B44] Dafoe A. Hughes E. Bachrach Y. Collins T. McKee K. R. Leibo J. Z. (2020). Open problems in cooperative AI. arXiv:2012.08630. Available online at: https://forum.effectivealtruism.org/posts/42reWndoTEhFqu6T8/ai-governance-opportunity-and-theory-of-impac

[B45] Dai Y. Zhang L. Yu J. Zhou X. He H. Ji Y.. (2021). Improved symptoms following bumetanide treatment in children aged 3– 6 years with autism spectrum disorder: a randomized, double-blind, placebo-controlled trial. Sci. Bullet. 66, 1591–1598. 10.1016/j.scib.2021.01.008 36654288

[B46] de Villiers J. (2007). The interface of language and theory of mind. Lingua 117, 1858–1878. 10.1016/j.lingua.2006.11.006 17973025 PMC2000852

[B47] de Villiers J. Hobbs K. Hollebrandse B. (2014). Recursive complements and propositional attitudes, in Recursion: Complexity in Cognition, ed T. Roeper and M. Speas (Cham: Springer), 221–242. 10.1007/978-3-319-05086-7_10

[B48] de Waal F. B. (2008). Putting the altruism back into altruism: the evolution of empathy. Annu. Rev. Psychol. 59, 279–300. 10.1146/annurev.psych.59.103006.093625 17550343

[B49] de Weerd H. Diepgrond D. Verbrugge R. (2018). Estimating the use of higher-order theory of mind using computational agents. BE J. Theoret. Econ. 18, 184. 10.1515/bejte-2016-0184

[B50] de Weerd H. Verbrugge R. Verheij B. (2017). Negotiating with other minds: the role of recursive theory of mind in negotiation with incomplete information. Auton. Agents Multi-Agent Syst. 31, 250–287. 10.1007/s10458-015-9317-1

[B51] Dempsey E. E. Moore C. Johnson S. A. Stewart S. H. Smith I. M. (2020). Morality in autism spectrum disorder: a systematic review. Dev. Psychopathol. 32, 1069–1085. 10.1017/S0954579419001160 31489833

[B52] Dimitrakakis C. Rothkopf C. A. (2011). Bayesian multitask inverse reinforcement learning. EWRL 2011: Recent Advances in Reinforcement Learning, 2011, 273–284. 10.1007/978-3-642-29946-9_27

[B53] D'mello S. Graesser A. (2013). AutoTutor and affective AutoTutor: learning by talking with cognitively and emotionally intelligent computers that talk back. ACM Transactions on Interactive Intelligent Systems 2, 1–39. 10.1145/2395123.2395128

[B54] Dunn J. Brophy M. (2005). Communication, relationships, and individual differences in children's understanding of mind, in Why Language Matters for Theory of Mind, Apr. 2002, Toronto, ON: University of Toronto. This chapter originated from the aforementioned conference. Oxford University Press. 10.1093/acprof:oso/9780195159912.003.0003

[B55] FeldmanHall O. Dalgleish T. Evans D. Mobbs D. (2015). Empathic concern drives costly altruism. Neuroimage 105, 347–356. 10.1016/j.neuroimage.2014.10.043 25462694 PMC4275572

[B56] Fickinger A. Zhuang S. Hadfield-Menell D. Russell S. (2020). Multi-principal assistance games. arXiv preprint arXiv:2007.09540. Available online at: https://arxiv.org/pdf/2007.09540.pdf

[B57] Finn C. Abbeel P. Levine S. (2017). Model-agnostic meta-learning for fast adaptation of deep networks, in Proceedings of the 34th International Conference on Machine Learning (Sydney, NSW).

[B58] Flavell J. H. Green F. L. Flavell E. R. Watson M. W. Campione J. C. (1986). Development of knowledge about the appearance-reality distinction. Monogr. Soc. Res. Child Dev. 1986, i*-*87. 10.2307/1165866 3807927

[B59] Flobbe L. Verbrugge R. Hendriks P. Krämer I. (2008). Children's application of theory of mind in reasoning and language. J. Logic Lang. Inform. 17, 417–442. 10.1007/s10849-008-9064-7

[B60] Frith C. Frith U. (2005). Theory of mind. Curr. Biol. 15, R644–R645. 10.1016/j.cub.2005.08.041 16139190

[B61] Frith U. Frith C. D. (2003). Development and neurophysiology of mentalizing. Philos. Trans. Royal Soc. Lond. Ser. B Biol Sci. 358, 459–473. 10.1098/rstb.2002.1218 12689373 PMC1693139

[B62] Gonzalez B. Chang L. J. (2021). Computational models of mentalizing, in The Neural Basis of Mentalizing (Cham: Springer), 299–315. 10.1007/978-3-030-51890-5_15

[B63] Gopnik A. Wellman H. M. (1992). Why the child's theory of mind really is a theory. Mind Lang. 7, 145–171. 10.1111/j.1468-0017.1992.tb00202.x24684559

[B64] Gordon R. M. (1986). Folk psychology as simulation. Mind Lang. 1, 158–171. 10.1111/j.1468-0017.1986.tb00324.x

[B65] Gordon R. M. (1996). ‘Radical' simulationism, in Theories of Theories of Mind, eds Carruthers, P. and Smith, P. K. (Cambridge: Cambridge University Press), 11–21. 10.1017/CBO9780511597985.003

[B66] Hadfield-Menell D. Dragan A. Abbeel P. Russell S. (2016). Cooperative inverse reinforcement learning, in NIPS'16: Proceedings of the 30th International Conference on Neural Information Processing Systems (Barcelona), 3916–3924.

[B67] Hadfield-Menell D. Dragan A. Abbeel P. Russell S. (2017a). The off-switch game, in IJCAI'17: Proceedings of the 26th International Joint Conference on Artificial Intelligence (Melbourne), 220–227. 10.24963/ijcai.2017/32

[B68] Hadfield-Menell D. Milli S. Abbeel P. Russell S. Dragan A. (2017b). Inverse reward design, in NIPS'17: Proceedings of the 31st International Conference on Neural Information Processing Systems (Long Beach, CA), 6768–6777.

[B69] Happé F. Brownell H. Winner E. (1999). Acquired theory of mind impairments following stroke. Cognition 70, 211–240. 10.1016/S0010-0277(99)00005-0 10384736

[B70] Harris J. Anthis J. R. (2021). The moral consideration of artificial entities: a literature review. Sci. Eng. Ethics 27, 53. 10.1007/s11948-021-00331-8 34370075 PMC8352798

[B71] Hedden T. Zhang J. (2002). What do you think I think you think? strategic reasoning in matrix games. Cognition 85, 1–36. 10.1016/S0010-0277(02)00054-9 12086711

[B72] Hiatt L. M. Trafton J. G. (2015). Understanding second-order theory of mind, in Proceedings of the Tenth Annual ACM/IEEE International Conference on Human-Robot Interaction Extended Abstracts (Portland), 167–168. 10.1145/2701973.2702030

[B73] Ho M. Littman M. MacGlashan J. Cushman F. Austerweil J. L. (2016). Showing versus doing: teaching by demonstration. Adv. Neural Inform. Proces. Syst. 29, 3035–3043. Available online at: https://papers.nips.cc/paper/2016/file/b5488aeff42889188d03c9895255cecc-Paper.pdf

[B74] Hong J. J. Milli S. Dragan A. (2020). Reward-rational (implicit) choice: a unifying formalism for reward learning, in Advances in Neural Information Processing Systems 33: Annual Conference on Neural Information Processing Systems, 2020.

[B75] Hughes C. Cutting A. L. (1999). Nature, nurture, and individual differences in early understanding of mind. Psychol. Sci. 10, 429–432. 10.1111/1467-9280.00181

[B76] Hughes C. Jaffee S. R. Happé F. Taylor A. Caspi A. Moffitt T. E. (2005). Origins of individual differences in theory of mind: from nature to nurture? Child Dev. 76, 356–370. 10.1111/j.1467-8624.2005.00850_a.x 15784087

[B77] Huguet G. Benabou M. Bourgeron T. (2016). *The Genetics of Autism Spectrum Disorders*.. A Time for Metabolism and Hormones. Cham: Springer, 101–129. 10.1007/978-3-319-27069-2_11 28892342

[B78] Jara-Ettinger J. (2019). Theory of mind as inverse reinforcement learning. Curr. Opin. Behav. Sci. 29, 105–110. 10.1016/j.cobeha.2019.04.010

[B79] Jonnavittula A. Losey D. P. (2021). I know what you meant: learning human objectives by (under)estimating their choice set, in IEEE International Conference on Robotics and Automation (ICRA) (Philadelphia). 10.1109/ICRA48506.2021.9562048

[B80] Kain W. Perner J. (2003). Do children with ADHD not need their frontal lobes for theory of mind? A review of brain imaging and neuropsychological studies. Soc. Brain Evol. Pathol. 10, 197–230. 10.1002/0470867221.ch10

[B81] Kanen J. W. Arntz F. E. Yellowlees R. Cardinal R. N. Price A. Christmas D. M.. (2021). Serotonin depletion amplifies distinct human social emotions as a function of individual differences in personality. Transl. Psychiatr. 11, 1–12. 10.1038/s41398-020-00880-9 33518708 PMC7847998

[B82] Kaplan J. McCandlish S. Henighan T. Brown T. B. Chess B. Child R. (2020). Scaling laws for neural language models. arXiv:2001.08361. Available online at: https://arxiv.org/pdf/2001.08361.pdf

[B83] Karnofsky H. (2021). Forecasting Transformative AI: the “Biological Anchors” Method in a Nutshell. Available online at: https://www.cold-takes.com/forecasting-transformative-ai-the-biological-anchors-method-in-a-nutshell/ (accessed September 1, 2021).

[B84] Kerr N. Dunbar R. I. Bentall R. P. (2003). Theory of mind deficits in bipolar affective disorder. J. Affect. Disord. 73, 253–259. 10.1016/S0165-0327(02)00008-3 12547294

[B85] Kim S. K. Kirchner E. A. Stefes A. Kirchner F. (2017). Intrinsic interactive reinforcement learning–using error-related potentials for real world human-robot interaction. Sci. Rep. 7, 1–16. 10.1038/s41598-017-17682-7 29242555 PMC5730605

[B86] Kim Y. S. G. (2020). Theory of mind mediates the relations of language and domain-general cognitions to discourse comprehension. J. Exp. Child Psychol. 194, 104813. 10.1016/j.jecp.2020.104813 32092536

[B87] Kinderman P. Dunbar R. Bentall R. P. (1998). Theory-of-mind deficits and causal attributions. Br. J. Psychol. 89, 191–204. 10.1111/j.2044-8295.1998.tb02680.x

[B88] Kumar R. Garrabrant S. (2019). Thoughts on Human Models. Available online at: https://www.alignmentforum.org/posts/BKjJJH2cRpJcAnP7T/thoughts-on-human-models (accessed September 1, 2021).

[B89] Lawrence A. Clark L. Labuzetta J. N. Sahakian B. Vyakarnum S. (2008). The innovative brain. Nature 456, 168–169. 10.1038/456168a 19005531

[B90] Le M. Boureau Y. L. Nickel M. (2019). Revisiting the evaluation of theory of mind through question answering, in Proceedings of the 2019 Conference on Empirical Methods in Natural Language Processing and the 9th International Joint Conference on Natural Language Processing (EMNLP-IJCNLP) (Hong Kong), 5872–5877. 10.18653/v1/D19-1598

[B91] Leslie A. M. Frith U. (1988). Autistic children's understanding of seeing, knowing and believing. Br. J. Dev. Psychol. 6, 315–324. 10.1111/j.2044-835X.1988.tb01104.x

[B92] Li J. Zhu L. Gummerum M. (2014). The relationship between moral judgment and cooperation in children with high-functioning autism. Sci. Rep. 4, 1–6. 10.1038/srep04314 24603775 PMC3945921

[B93] Lindner D. Shah R. Abbeel P. Dragan A. (2021). Learning what to do by simulating the past, in International Conference on Learning Representations (ICLR), 2021.

[B94] Liszkowski U. Carpenter M. Striano T. Tomasello M. (2006). 12-and 18-month-olds point to provide information for others. J. Cogn. Dev. 7, 173–187. 10.1207/s15327647jcd0702_2

[B95] Mars R. B. Neubert F. X. Noonan M. P. Sallet J. Toni I. Rushworth M. F. (2012). On the relationship between the “default mode network” and the “social brain. Front. Hum. Neurosci. 6, 189. 10.3389/fnhum.2012.00189 22737119 PMC3380415

[B96] McAlister A. Peterson C. C. (2006). Mental playmates: siblings, executive functioning and theory of mind. Bri. J. Dev. Psychol. 24, 733–751. 10.1348/026151005X70094

[B97] Meijering B. Taatgen N. A. van Rijn H. Verbrugge R. (2014). Modeling inference of mental states: as simple as possible, as complex as necessary. Interact. Stud. 15, 455–477. 10.1075/is.15.3.05mei

[B98] Meijering B. Van Maanen L. Van Rijn H. Verbrugge R. (2010). The facilitative effect of context on second-order social reasoning, in Proceedings of the Annual Meeting of the Cognitive Science Society (Portland), 32.

[B99] Mendez J. Shivkumar S. Eaton E. (2018). Lifelong inverse reinforcement learning, in Advances in Neural Information Processing Systems (Montreal), 31.

[B100] Mier D. Lis S. Neuthe K. Sauer C. Esslinger C. Gallhofer B.. (2010). The involvement of emotion recognition in affective theory of mind. Psychophysiology 47, 1028–1039. 10.1111/j.1469-8986.2010.01031.x 20456660

[B101] Mindermann S. Armstrong S. (2018). Occam's razor is insufficient to infer the preferences of irrational agents, in Proceedings of the 32nd International Conference on Neural Information Processing Systems (NIPS'18). Red Hook, NY: Curran Associates Inc., 5603–5614.

[B102] Moor J. H. (2011). The nature, importance, and difficulty of machine ethics. Machine Ethics 3, 13–20. 10.1017/CBO9780511978036.003

[B103] Moreno P. Hughes E. McKee K. R. Pires B. A. Weber T. (2021). Neural recursive belief states in multi-agent reinforcement learning. arXiv preprint arXiv:2102.02274. Available online at: https://arxiv.org/abs/2102.02274

[B104] Nematzadeh A. Burns K. Grant E. Gopnik A. Griffiths T. L. (2018). Evaluating theory of mind in question answering. arXiv preprint arXiv:1808.09352. 10.18653/v1/D18-1261

[B105] Ng A. Y. Russell S. (2000). Algorithms for inverse reinforcement learning, in Proc. 17th International Conf. on Machine Learning (Stanford, CA), 663–670.

[B106] Ong D. C. Zaki J. Goodman N. D. (2019). Computational models of emotion inference in theory of mind: a review and roadmap. Top. Cogn. Sci. 11, 338–357. 10.1111/tops.12371 30066475 PMC7077035

[B107] Padmanabhan A. Lynch C. J. Schaer M. Menon V. (2017). The default mode network in autism. Biol. Psychiatr. 2, 476–486. 10.1016/j.bpsc.2017.04.004 29034353 PMC5635856

[B108] Pavăl D. (2017). A dopamine hypothesis of autism spectrum disorder. Dev. Neurosci. 39, 355–360. 10.1159/000478725 28750400

[B109] Phillips L. H. MacLean R. D. Allen R. (2002). Age and the understanding of emotions: neuropsychological and sociocognitive perspectives. J. Gerontol. Ser. B Psychol. Sci. Soc. Sci. 57, P526–P530. 10.1093/geronb/57.6.P526 12426435

[B110] Phillips W. Baron-Cohen S. Rutter M. (1998). Understanding intention in normal development and in autism. Br. J. Dev. Psychol. 16, 337–348. 10.1111/j.2044-835X.1998.tb00756.x

[B111] Pratt C. Bryant P. (1990). Young children understand that looking leads to knowing (so long as they are looking into a single barrel). Child Dev. 61, 973–982. 10.2307/1130869 2209200

[B112] Premack D. Woodruff G. (1978). Does the chimpanzee have a theory of mind? Behav. Brain Sci. 1, 515–526. 10.1017/S0140525X00076512

[B113] Rabinowitz N. Perbet F. Song F. Zhang C. Eslami S. M. A. Botvinick M. (2018). Machine theory of mind, in Proceedings of the 35th International Conference on Machine Learning, PMLR (80) (Stockholm), 4218–4227.

[B114] Ramachandran D. Amir E. (2007). Bayesian inverse reinforcement learning, in IJCAI'07: Proceedings of the 20th International Joint Conference on Artificial Intelligence (Hyderabad), 2586–2591.

[B115] Reddy S. Dragan A. Levine S. (2018). Where do you think you're going? inferring beliefs about dynamics from behavior, in Proceedings of the 32nd International Conference on Neural Information Processing Systems (NIPS'18). Red Hook, NY: Curran Associates Inc., 1461–1472.

[B116] Roiser J. P. Sahakian B. J. (2013). Hot and cold cognition in depression. CNS Spectrums 18, 139–149. 10.1017/S1092852913000072 23481353

[B117] Rusch T. Steixner-Kumar S. Doshi P. Spezio M. Jan Gläscher J. (2020). Theory of mind and decision science: towards a typology of tasks and computational models. Neuropsychologia. 146, 107488. 10.1016/j.neuropsychologia.2020.107488 32407906

[B118] Russell S. (2019). Human Compatible: Artificial Intelligence and the Problem of Control. London: Penguin UK.

[B119] Sally D. Hill E. (2006). The development of interpersonal strategy: autism, theory-of-mind, cooperation and fairness. J. Econ. Psychol. 27, 73–97. 10.1016/j.joep.2005.06.015

[B120] Sap M. Rashkin H. Chen D. LeBras R. Choi Y. (2019). Socialiqa: commonsense reasoning about social interactions. arXiv preprint arXiv:1904.09728. 10.18653/v1/D19-1454

[B121] Saxe R. Carey S. Kanwisher N. (2004). Understanding other minds: linking developmental psychology and functional neuroimaging. Annu. Rev. Psychol. 55, 87–124. 10.1146/annurev.psych.55.090902.142044 14744211

[B122] Schlaffke L. Lissek S. Lenz M. Juckel G. Schultz T. Tegenthoff M.. (2015). Shared and nonshared neural networks of cognitive and affective theory-of-mind: a neuroimaging study using cartoon picture stories. Hum. Brain Map. 36, 29–39. 10.1002/hbm.22610 25131828 PMC6869702

[B123] Schurz M. Radua J. Aichhorn M. Richlan F. Perner J. (2014). Fractionating theory of mind: a meta-analysis of functional brain imaging studies. Neurosci. Biobehav. Rev. 42, 9–34. 10.1016/j.neubiorev.2014.01.009 24486722

[B124] Senju A. Southgate V. Miura Y. Matsui T. Hasegawa T. Tojo Y.. (2010). Absence of spontaneous action anticipation by false belief attribution in children with autism spectrum disorder. Dev. Psychopathol. 22, 353–360. 10.1017/S0954579410000106 20423546

[B125] Sensoy M. Kaplan L. Kandemir M. (2018). Evidential deep learning to quantify classification uncertainty. arXiv preprint arXiv:1806.01768. Available online at: https://arxiv.org/abs/1806.01768

[B126] Shah R. Freire P. Alex N. Freedman R. Krasheninnikov D. Chan L. (2021a). Benefits of Assistance Over Reward Learning. Available online at: https://openreview.net/forum?id=DFIoGDZejIB (accessed September 1, 2021).

[B127] Shah R. Gundotra N. Abbeel P. Dragan A. (2019a). On the feasibility of learning, rather than assuming, human biases for reward inference, in International Conference on Machine Learning (Long Beach, CA), 5670–5679.

[B128] Shah R. Krasheninnikov D. Alexander J. Abbeel P. Dragan A. (2019b). Preferences implicit in the state of the World, in International Conference on Learning Representations (ICLR) (New Orleans), 2019.

[B129] Shah R. Wild C. Wang S. H. Alex N. Houghton B. Guss W. (2021b). The MineRL BASALT competition on learning from human feedback. arxiv:2107.01969. Available online at: https://arxiv.org/abs/2107.01969

[B130] Slivkins A. (2019). Introduction to multi-armed bandits. Found. Trends Machine Learn. 12, 1–286. 10.1561/9781680836219

[B131] Southgate V. Senju A. Csibra G. (2007). Action anticipation through attribution of false belief by 2-year-olds. Psychol. Sci. 18, 587–592. 10.1111/j.1467-9280.2007.01944.x 17614866

[B132] Spindler L. R. Luppi A. I. Adapa R. M. Craig M. M. Coppola P. Peattie A. R.. (2021). Dopaminergic brainstem disconnection is common to pharmacological and pathological consciousness perturbation. Proc. Natl. Acad. Sci. U. S. A. 118:pnas.2026289118. 10.1073/pnas.2026289118 34301891 PMC8325270

[B133] Sutton R. (2019). The Bitter Lesson. Available online at: http://www.incompleteideas.net/IncIdeas/BitterLesson.html (accessed September 1, 2021).

[B134] Trafton J. G. Hiatt L. M. Harrison A. M. Tamborello F. P. Khemlani S. S. Schultz A. C. (2013). Act-r/e: an embodied cognitive architecture for human-robot interaction. J. Hum. Robot Interact. 2, 30–55. 10.5898/JHRI.2.1.Trafton

[B135] Uljarevic M. Hamilton A. (2013). Recognition of emotions in autism: a formal meta-analysis. J. Autism Dev. Disord. 43, 1517–1526. 10.1007/s10803-012-1695-5 23114566

[B136] Van de Ven G. M. Tolias A. S. (2019). Three scenarios for continual learning. arXiv preprint arXiv:1904.07734. Available online at: https://arxiv.org/abs/1904.07734

[B137] Vanschoren J. (2018). Meta-learning: a survey. arXiv preprint arXiv:1810.03548. Available online at: https://arxiv.org/abs/1810.03548

[B138] Veltman K. de Weerd H. Verbrugge R. (2019). Training the use of theory of mind using artificial agents. J. Multimodal User Interfaces 13, 3–18. 10.1007/s12193-018-0287-x

[B139] Völlm B. A. Taylor A. N. Richardson P. Corcoran R. Stirling J. McKie S.. (2006). Neuronal correlates of theory of mind and empathy: a functional magnetic resonance imaging study in a nonverbal task. Neuroimage 29, 90–98. 10.1016/j.neuroimage.2005.07.022 16122944

[B140] Wellman H. M. Estes D. (1986). Early understanding of mental entities: a reexamination of childhood realism. Child Dev. 1986, 910–923. 10.2307/1130367 3757609

[B141] Wen Y. Yang Y. Luo R. Wang J. Pan W. (2019). Probabilistic recursive reasoning for multi-agent reinforcement learning. arXiv preprint arXiv:1901.09207. Available online at: https://arxiv.org/abs/1901.09207

[B142] Westby C. Robinson L. (2014). A developmental perspective for promoting theory of mind. Top. Lang. Disord. 34, 362–383. 10.1097/TLD.0000000000000035

[B143] Wimmer H. Perner J. (1983). Beliefs about beliefs: representation and constraining function of wrong beliefs in young children's understanding of deception. Cognition 13, 103–128. 10.1016/0010-0277(83)90004-5 6681741

[B144] Woodward M. Finn C. Hausman K. (2020). Learning to interactively learn and assist. Proc. AAAI Conference Artif. Intelligen. 34, 2535–2543. 10.1609/aaai.v34i03.5636

[B145] Xu K. Ratner E. Dragan A. Levine S. Finn C. (2019). Learning a prior over intent via meta-inverse reinforcement learning. Proc. 36th Int. Conference Machine Learn. 97, 6952–6962. Available online at: https://arxiv.org/abs/1805.12573

[B146] Yoshida W. Dolan R. J. Friston K. J. (2008). Game theory of mind. PLoS Comput. Biol. 4, e1000254. 10.1371/journal.pcbi.1000254 19112488 PMC2596313

[B147] Zellers R. Holtzman A. Clark E. Qin L. Farhadi A. Choi Y. (2021). Turing advice: a generative and dynamic evaluation of language use, in Proceedings of the 2021 Conference of the North American Chapter of the Association for Computational Linguistics: Human Language Technologies, Association for Computational Linguistics, 4856-4880. 10.18653/v1/2021.naacl-main.386

[B148] Zeng Y. Zhao Y. Zhang T. Zhao D. Zhao F. Lu E. (2020). A brain-inspired model of theory of mind. Front. Neurorobot. 14, 60. 10.3389/fnbot.2020.00060 32982714 PMC7483660

[B149] Zhang L. Huang C. C. Dai Y. Luo Q. Ji Y. Wang K.. (2020). Symptom improvement in children with autism spectrum disorder following bumetanide administration is associated with decreased GABA/glutamate ratios. Transl. Psychiatr. 10, 1–12. 10.1038/s41398-020-0692-2 32066666 PMC7026137

[B150] Zhang S. (2019). Summary of Recommender Systems Surveys in Recent Years. Available online at: https://shuaizhang.tech/posts/2019/08/blog-post-2/ (accessed December 13, 2021).

[B151] Ziebart B. D. Andrew J. Carnegie B. (2010). Modeling interaction *via* the principle of maximum causal entropy, in Proceedings of the Twenty-seventh International Conference on Machine Learning (Chicago), 1255–1262.

[B152] Ziebart B. D. Maas A. Bagnell J. A. Dey A. K. (2008). Maximum entropy inverse reinforcement learning, in AAAI'08: Proceedings of the 23rd National Conference on Artificial Intelligence (Haifa), 1433–1438.

